# Surveillance study of species distribution, antifungal susceptibility and mortality of nosocomial candidemia in a tertiary care hospital in China

**DOI:** 10.1186/1471-2334-13-337

**Published:** 2013-07-22

**Authors:** Chun-fang Ma, Fang-qiu Li, Li-ning Shi, Yu-an Hu, Ying Wang, Mei Huang, Qian-qian Kong

**Affiliations:** 1 Laboratory of Molecular Biology, Institute of Medical Laboratory Sciences, Jinling Hospital, School of Medicine, Nanjing University, Nanjing 210002, PR China; 2 Laboratory of Molecular Biology, Institute of Medical Laboratory Sciences, Jinling Hospital, 305 East Zhongshan Road, Nanjing 210002, P. R. China

**Keywords:** Nosocomial candidemia, Antifungal susceptibility, Mortality, Risk factors

## Abstract

**Background:**

Bloodstream infections due to *Candida* species cause significant morbidity and mortality, and the epidemiology of *Candida* infection is changing. Surveillance for candidemia is necessary to detect trends in species distribution and antifungal resistance.

**Methods:**

The medical and electronic records of all patients who had candidemia at the authors’ hospital from 2009 to 2011 were reviewed for demographic data and clinical information, including the infecting *Candida* species, resistance to antifungals and survival, and the presence of risk factors associated with candidemia.

**Results:**

A total of 133 distinct episodes of candidemia were identified over the study period. The annual incidence of candidemia ranged between 0.71 and 0.85 cases/1000 hospital discharges. The most frequent *Candida* species were *C. tropicalis* (28.6%), followed by *C. albicans* (23.3%) and *C. parapsilosis* (19.5%). The rates of susceptibility to antifungal agents were as followed: voriconazole (97.8%), itraconazole (69.5%), fluconazole (46.1%), ketoconazole (38.9%). Out of 131 evaluable patients, 34 (26.0%) died within 30 days from the onset of candidemia. *C. tropicalis* candidemia was associated with the highest mortality rate (44.7%). Regarding the crude mortality in the different units, patients in Hemato-Oncology ward had the highest mortality rate (66.7%), followed by patients in cardiovascular wards and ICU (57.1% and 25.6%, respectively). Predictors of 30-day mortality were identified by uni- and multivariate analyses. Complicated abdominal surgery, presence of central venous catheter (CVC), neutropenia, candidemia due to *C. tropicalis* and poor treatment with fluconazole were significantly associated with the 30-day mortality. Presence of CVC (odds ratio[OR] = 4.177; 95% confidence interval [CI] = 1.698 to 10.278; *P* = 0.002) was the only independent predictor for mortality in the multivariate analysis.

**Conclusion:**

This report provides baseline data for future epidemiological and susceptibility studies and for the mortality rates associated with candidemia in our hospital. The knowledge of the local epidemiological trends in *Candida* species isolated in blood cultures is important to guide therapeutic choices.

## Background

*Candida* spp. is one of the most frequent pathogens isolated in bloodstream infections (BSI), and is associated with significant morbidity and mortality [1,2]. During the past two decades, the incidence of candidemia has been doubled and *Candida* spp. currently ranks as the fourth and the seventh most common bloodstream pathogen in North American and European studies [3,4]. The reasons for the increasing candidemia rates include improved detection as well as increase in patient-population at risk, such as invasive procedures and devices, broad-spectrum antimicrobial agents, advanced life-support and aggressive chemotherapy are more extensively used [5].

Candidemia have been associated with high crude and attributable mortality rates, especially among critically ill patients. The crude mortality rate of these infections is 40%-75% and the attributable mortality of candidemia has been estimated at 25%-38% [6,7]. In addition, an increase of 30 days in the length of hospital stay among patients surviving these infections has been demonstrated [8]. The economic impact of these infections is also important: candidemia has been associated with increased costs of care and prolonged hospitalization [9].

Historically, *Candida albicans* is the most common cause of candidemia worldwide. However, in recent years, some studies have reported an increase of candidemia due to non-*albicans Candida* species, with the threat of increased mortality and antifungal drug resistance [10,11]. The intrinsic and emerging resistance to azoles actually represents a major challenge for empirical therapeutic and prophylactic strategies [12].

The epidemiology of candidemia shows a wide variation among different countries. For example, an increasing incidence of candidemia in Iceland was reported for the period between 2000 and 2011 [13], but the similarity was not observed in Switzerland, where a national surveillance study showed that the incidence of candidemia had remained unchanged during the period of 1991 to 2000 [4]. Despite the epidemiology of candidemia has been studied extensively in the United States, Europe, and some countries in South America, very few studies have addressed these issues in China, where the differences about the demographic characteristics, health care practices, patterns using blood cultures, and antibiotic usage as well as the resistance situation do exist compared to other countries. In the present study, a three-year retrospective analysis was conducted to evaluate the incidence, species distribution, the associated resistance patterns of *Candida* species for the contemporary azole antifungal agents, outcome of candidema BSI and risk factors for mortality in patients with candidemia in Jinling hospital, Nanjing, China.

## Methods

### Collection of patients

In our previous study, we developed two enzyme-linked immunosorbent assays to detect specific antibodies against *Candida* Eno and Fba1, and investigated the diagnostic value for invasive *Candida* infections by analyzing sera from patients with candidemia [14]. The purpose of current study was to investigate the state of *Candida* invasive infection. We conducted a retrospective observational study of computerized laboratory records of positive blood cultures at Jinling Hospital (Nanjing, China), a 1,800 beds tertiary care hospital with about 55,000 admissions per year from January 2009 to December 2011. The target population consisted of hospitalized patients presenting risk factors for candidemia, especially patients receiving broad-spectrum antibiotics, immunosuppressive therapy, parenteral nutrition, abdominal/thoracic surgery and hematopoietic transplantation, or those patients who had a long intensive care unit stay or acute renal failure. An episode of candidemia was defined as the isolation of a *Candida* species from blood culture in a patient with temporally related clinical signs and symptoms, such as fever, chills, or hypotension. For each patient, only the first episode of candidemia was included. Patients whose cultures grew >1 species of *Candida* were excluded from the analysis and all data were collected by using the electronic medical records. The following data were retrospectively collected: basic demographics, underlying disease, the specific fungal pathogen and species, resistance to antifungals and survival. The presence of risk factors associated with candidemia were also collected: systemic corticosteroid treatment (a dose equivalent to prednisone 10 mg/d for at least 14 days), neutropenia (absolute neutrophil count <500 cells/μl), complicated abdominal surgery (severe acute pancreatitis, and complex ventral hernia), acute renal failure (sudden and often temporary loss of kidney function with nitrogen waste retention and hypourocrinia), indwelling central vascular catheter, intensive care unit hospitalization or mechanical ventilation. Early mortality was defined as death within seven days and late mortality as death between seven and 30 days [15]. The study protocol was approved by the Medical Ethics Committee of Jinling hospital.

### Organism identification

A volume of 3 ml blood for children and 5 ~ 10 ml blood for adults was collected aseptically by skin venipuncture taken from different parts of each patient. The blood was inoculated into both BacT/AlerT 3D aerobic and anaerobic vials (Becton Dickinson). All positive cultures were manually sampled and inoculated on CHROMagar Candida medium (CHROMagar Company, Paris France) to ensure viability and purity. An aliquot was Gram-stained for preliminary identification of the microorganism. Identification of all species was confirmed with the Vitek-2 system (bioMerieux, France).

### Agar diffusion method

The growth inhibition zone and the category (resistant, intermediate and susceptible) were determined for fluconazole (FLC), itraconazole (ITC), ketoconazole (KTC), voriconazole (VOR), using a commercial agar diffusion test with Neo-Sensitabs® tablets (Rosco, Denmark). Neo-Sensitabs® sensitivity testing is a standardized agar diffusion method which includes antifungal agents in tablets of 9 mm of diameter with FLC (25 μg), ITC (8 μg), KTC (15 μg) and VOR (1 μg). Neo-Sensitabs® assay was performed according to the manufacturer’s instructions [16]. Inocula contained 5 × 10^5^ cells/ml and were prepared from an overnight subculture on Sabouraud glucose agar (Difco, USA), getting suspensions corresponding to McFarland 0.5 standard and then diluted (1:1) with sterile saline solutions. For *C. krusei* isolates inocula were equivalent to McFarland 0.5 standard, diluted 1:10 in saline solutions. Two milliliters of each inoculum suspensions were poured onto the agar surface and later removing the liquid in excess. Opened plates were dried at 35°C for 15 min before tablets will be placed on the agar surface.

Shadomy modified medium was used (Yeast Nitrogen Base, asparagine and glucose) with phosphate buffer to pH 7 and containing chloramphenicol to avoid bacterial contamination. Media was heated at 100°C during 15 min, and then cooled to 60°C before pouring the medium in Petri dishes (12 cm × 12 cm). A pre-diffusion drying was made at 37°C during 15 min. After an incubation period of 20-24 h, the inhibition diameter zones were read in mm with a caliper (Mitutoyo, Japan). Zone diameters were measured to the nearest whole millimeter at a point in which there was a prominent reduction of growth (80% for azoles). Depending on the diameter of the inhibition zone, *Candida* strains were classified as susceptible (S), intermediate (I) or resistant (R) to tested antifungals. The basic criterion according to manufacturers instructions was as follows: FLC (S ≥ 19 mm, I18~15 mm, R ≤ 14 mm); ITC (S ≥ 15 mm, I14~10 mm, R < 10 mm); KTC (S ≥ 28 mm, I27~21 mm, R ≤ 20 mm); VOR (S ≥ 17 mm, I16~14 mm, R ≤ 13 mm).

### Statistical analyses

In the assessment of predictors of poor outcome, we compared patients who survived until day 30 after the incident candidemia with those who died. Univariate analysis was perforemd using Fisher exact test or Chi-squared test for categorical variables. All variables with *P*-value < 0.1 by univariate analysis were entered in a logistic regression model. Kaplan-Meier curves were constructed to assess survival, and curve comparisons were performed by the Log Rank test. All tests were two-tailed and a level of significance of *P* < 0.05 was considered statistically significant. Statistical analysis was performed using SPSS 18.0 for Windows (SPSS, Chicago, IL, USA).

## Results

A total of 133 distinct episodes of candidemia (130 adults and 3 children <15 years of age) were identified during the study period. Most candidemia was reported in adults (97.7%). More candidemia occurred among males (68.4%) than females, mainly in those over 60 years of age (Table 1). The mean annual incidence of candidaemia was 0.77 per 1000 admission. The incidence of candidemia increased from 0.71 in 2009 to 0.85 episodes/1000 admission in 2011. But the change in incidence over time did not reach statistical significance. The demographic and clinical characteristics of the patients are summarized in Table 1. Most patients had one or more comorbidity at the time of the diagnosis of candidaemia. The most common underlying conditions documented prior to candidemia were intestinal fistula/abdominal infection (55.7%), respiratory dysfunction (12.8%), and gastrointestinal pathology (10.2%).

**Table 1:** Patient characteristics and incidence (episode/1000 admission)

	***Candida *species**							
	**Total**	***C. tropicalis***	***C. albicans***	***C. parapsilosis***	***C. glabrata***	***C. krusei***	***C. guilliermondii***	Other^*^
	**(n = 133)**	**(n = 38)**	**(n = 31)**	**(n = 26)**	**(n = 11)**	**(n = 3)**	**(n = 6)**	**(n = 18)**
	**100.0%**	**28.6%**	**23.3%**	**19.5%**	**8.3%**	**2.3%**	**4.5%**	**13.5%**
Patients characteristic								
Age (years)								
1-14	3(2.3)	1(33.3)	1(33.3)	0(0)	0(0)	0(0)	0(0)	1(33.3)
15-49	29(21.8)	7(24.1)	9(31.0)	5(17.2)	3(10.3)	0(0)	1(3.4)	4(13.8)
50-65	40(30.1)	13(32.5)	7(17.5)	8(20.0)	3(7.5)	1(2.5)	4(10.0)	4(10.0)
>65	61(45.9)	17(27.9)	14(23.0)	13(21.3)	5(8.2)	2(3.3)	1(1.6)	9(14.8)
Gender								
Male	91(68.4)	28(30.8)	20(22.0)	18(19.8)	6(6.6)	2(2.2)	2(2.2)	15(16.5)
Female	42(31.6)	10(23.8)	11(26.2)	8(19.0)	5(11.9)	1(2.4)	4(9.5)	3(7.1)
Underline diseases *n *(%)								
Intestinal fistula/abdominal infection	74(55.7)	14(18.9)	20(27.0)	15(20.3)	6(8.1)	3(4.1)	5(6.8)	11(14.9)
Solid tumor	8(6.0)	2(25.0)	2(25.0)	1(12.5)	2(25.0)	0(0)	0(0)	1(12.5)
Respiratory dysfunction^a^	17(12.8)	11(64.7)	3(17.6)	1(5.9)	0(0)	0(0)	0(0)	2(11.8)
Gastrointestinal pathology^b^	14(10.5)	4(28.6)	1(7.1)	5(35.7)	1(7.1)	0(0)	1(7.1)	2(14.3)
Haematological malignancy	6(4.5)	5(83.3)	1(16.7)	0(0)	0(0)	0(0)	0(0)	0(0)
Acute renal failure^c^	11(8.3)	1(9.1)	3(27.3)	4(36.4)	2(18.2)	0(0)	0(0)	1(9.1)
Burns	3(2.3)	1(33.3)	1(33.3)	0(0)	0(0)	0(0)	0(0)	1(33.3)
Receipt of corticosteroids	76(57.1)	21(27.6)	17(22.4)	15(19.7)	5(6.6)	1(1.3)	2(2.6)	15(19.7)
Chemotherapy	6(4.5)	5(83.3)	1(16.7)	0(0)	0(0)	0(0)	0(0)	0(0)
Presence of CVC^d^	61(45.9)	19(31.1)	13(21.3)	11(18.0)	5(8.2)	2(3.3)	4(6.6)	7(11.5)
Parenteral nutrition	61(45.9)	20(32.8)	18(29.5)	6(9.8)	8(13.1)	1(1.6)	3(4.9)	5(8.2)
Mechanical ventilation	17(12.8)	6(35.3)	5(29.4)	2(11.8)	2(11.8)	0(0)	0(0)	2(11.8)
In the ICU at diagnosis	39(29.3)	14(35.9)	9(23.1)	8(20.5)	3(7.7)	2(5.1)	0(0)	3(7.7)
Neutropenia^e^	6(4.5)	2(33.3)	2(33.3)	0(0)	2(33.3)	0(0)	0(0)	0(0)
Dialysis	2(1.5)	0(0)	1(50.0)	1(50.0)	0(0)	0(0)	0(0)	0(0)
Exposition to antibiotics	80(60.2)	29(36.3)	23(28.8)	20(25.0)	3(3.8)	2(2.6)	1(1.3)	2(2.6)
Treatment with fluconazole	61(45.9)	23(37.7)	18(29.5)	5(8.2)	3(4.9)	3(4.9)	2(3.3)	7(11.5)
Incidence (episodes/1000 admissions)								
2009	0.71	0.19	0.22	0.11	0.06	0.02	0.04	0.06
2010	0.77	0.18	0.15	0.23	0.02	0.03	0.03	0.15
2011	0.85	0.31	0.19	0.13	0.13	0	0.03	0.12

^*^Others include: *C. pelliculosa*(8)*, C. famata*(7) and *C. haemulonii*(2)*.*

^a^Includes the following diseases: pneumonia, chronic obstructive pulmonary disease, and acute respiratory distress syndrome.

^b^Includes the following diseases: cholecystitis, pancreatitis, peritonitis, and hepatitis.

^c^Acute renal failure was defined as sudden and often temporary loss of kidney function with nitrogen waste retention and hypourocrinia.

^d^*CVC* central venous catheter.

^e^Neutropenia was defined as absolute neutrophil count <500 cells/μl.

The percentages of the three most common *Candida* spp. isolated were as follows: *C. tropicalis* (28.6%), *C. albicans* (23.3%) and *C. parapsilosis* (19.5%) (Table 1). The causative organism varied according to age of patients and their underlying diseases. With increasing age, a reduction in the percentage of *C. albicans* (from 33.3% to 23%) and *C. tropicalis* (from 33.3% to 27.9%) and a significant increase of *C. parapsilosis* (from 0 to 21.3%) was observed. Non-*albicans Candida* species were isolated with the highest frequency from patients with respiratory dysfunction (82.4%) and haematological malignancies (83.3%), in which *C. tropicalis* was the predominant species (64.7% and 83.3%, respectively).

**Figure 1:**
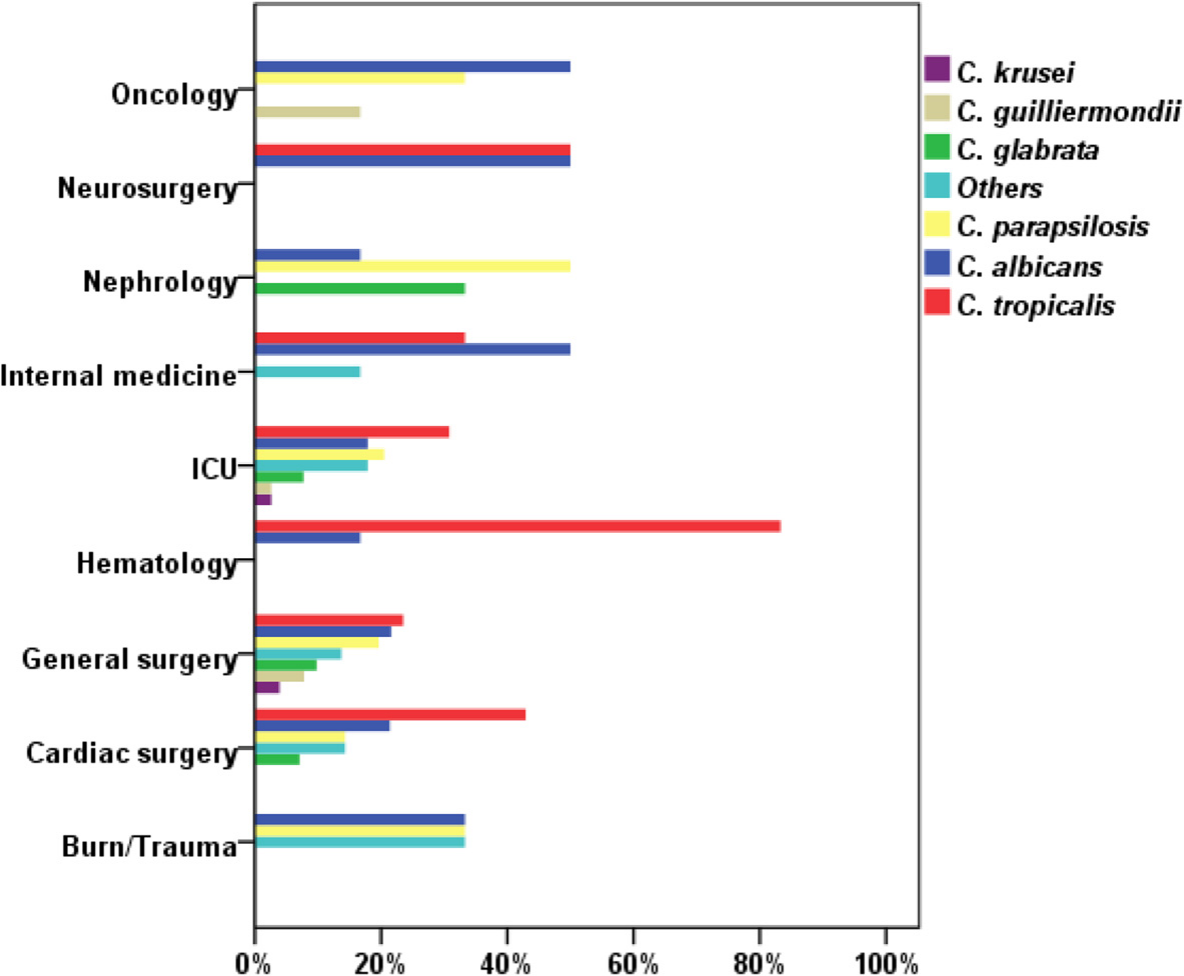
**Distribution of the *Candida *species according to underlying pathology/medical care (n).**

The distribution of isolated *Candida* species is shown in Figure 1. Globally, the high rates of candidemia infection are not limited to ICU but also occurred on general wards. Out of 133 evaluable patients, 51 (38.3%) were hospitalized in ward of general surgery, this proportion being a small amount higher than that which arose from patients in ICU (23.3%). The distribution of *Candida albicans* and non-*albicans Candida* species differed according to the type of patient population and risk factors, as shown in Figure 1. In patients with hematologic malignancies, *C. albicans* accounted for 16.7% of the cases and *C. tropicalis* for 83.3%; In ICU *C. albicans* was isolated in 17.9% of the cases, *C. tropicalis* in 30.8% and *C. parapsilosis* in 20.5%; on the other hand in oncology *C. albicans* was isolated in 50% of the cases.

Table 2 summarizes the results of in vitro susceptibility testing of bloodstream isolates of *Candida* in the study. Consistent with previous reports [17,18], vorconazole demonstrated excellent in vitro potency against *Candida* species, nearly all strains (97.8%) were susceptible to VOR. With regard to KTC, the rate of resistant is particularly high in *Candida* spp. (61.9%), almost all isolates were resistant to KTC. ITC resistance was observed for 30.5% of all *Candida* isolates evaluated and was also highest among *C. glabrata* isolates (90.9% resistance). Most strains (53.9%) became resistant to FLC, only 8 *C. albicans* BSI isolates (25.8%) collected during the 3-year surveillance program were reported as resistant. Decreased susceptibility to FLC was mostly seen with *C. glabrata* (90.9% resistance) and *C. tropicalis* (68.4% resistance). The percentages of yeast isolates with resistance and decreased susceptibility to FLC were similar during the three phases.

**Table 2:** **Antifungal susceptibility of *Candida *bloodstream isolates in patients during the study period**

	**Fluconazole**		**Itraconazole**		**Ketoconazole**		**Voriconazole**	
**Species**	**No. of isolates tested**	**%S**^**#**^	**No. of isolates tested**	**%S**	**No. of isolates tested**	**%S**	**No. of isolates tested**	**%S**
*C. albicans*	31	23 (74.2)	31	29 (93.6)	6	3 (50.0)	22	22 (100.0)
*C. parapsilosis*	26	15 (57.7)	26	25 (96.2)	2	1 (50.0)	22	22 (100.0)
*C. glabrata*	11	1 (9.1)	11	1 (9.1)	1	0 (0)	5	5 (100.0)
*C. tropicalis*	38	12 (31.6)	38	25 (65.8)	5	0 (0)	23	21 (91.3)
*C. krusei*	3	0 (0)	3	1 (33.3)	1	0 (0)	3	3 (100.0)
*C. guilliermondii*	6	4 (66.7)	6	5 (83.3)	0	0 (0)	5	5 (100.0)
Other^*^	16	4 (25.0)	16	5 (31.3)	3	3 (100.0)	12	12 (100.0)
Total	128	59 (46.1)	131	91 (69.5)^*^	18	7 (38.9)	92	90 (97.8)

*; Others include: *C. pelliculosa, C. famata* and *C. haemuloni.*

#; Abbreviations: *S* susceptible.

Outcome at day 30 was available for 131 episodes; patients in the remaining 2 episodes were lost to follow up. The crude mortality rate was 26.0%; Seven patients died within 48 hours of obtaining the blood culture, the time by which positive blood culture reports became available. This overall mortality rate varied according to the patients with different species of *Candida*, as well as the underlying disease or condition, the highest mortality rate was observed for *C. tropicalis* fungemia (44.7%). Haematological malignancy (66.7%), cardiovascular disease (57.1%), and intensive care in adults (25.6%) were associated more frequently with death during the study period.

Univariate predictors of poor outcome in candidemia is shown in Table 3. For candidemic patients, the variables associated with 30-day mortality were as follows: complicated abdominal surgery, presence of CVC, neutropenia, candidemia due to *C. tropicalis*. Major factor associated with 30-day survival was receipt of fluconazole as primary treatment for candidemia. Because neutropenia was associated with poor outcome and this variable was available in only 6 of the 131 patients, this variable was not included in the multiply logistic regression model. As shown in Table 4, predictors of 30-day mortality by multivariate analysis among patients with candidemia were presence of CVC (OR 4.177; 95% CI 1.698-10.278; *P* = 0.002).

**Table 3:** Factors associated with 30-day mortality by univariate analysis in candidemic patients

	**30-day outcome**		
**Variable**	**Survived N = 97**	**Died N = 34**	***P*-value**
Median age, years (range)	61 (0-88)	69 (0-73)	0.58
Gender (male:female)	68:29	23:11	0.889
In the ICU at diagnosis, *n* (%)	30 (30.9)	9 (26.5)	0.457
Mechanical ventilation, *n* (%)	10 (10.3)	7 (20.6)	0.109
Parenteral nutrition, *n* (%)	43 (44.3)	18 (52.9)	0.952
Complicated abdominal surgery, *n* (%)	21 (21.6)	14 (41.2)	0.019
Presence of CVC, *n* (%)	34 (35.1)	27 (79.4)	<0.001
Receipt of corticosteroids, *n* (%)	53 (54.6)	23 (67.6)	0.397
Neutropenia, *n* (%)	2 (2.1)	4 (11.8)	0.015
Species, *n* (%)			
*C. albicans*	25 (25.8)	6 (17.6)	0.508
*C. tropicalis*	21 (21.6)	17 (50.0)	0.002
*C. parapsilosis*	19 (19.6)	5 (14.7)	0.444
*C. glabrata*	8 (8.2)	3 (8.8)	0.928
*C. krusei*	2 (2.1)	1 (2.9)	0.575
Treatment with antibiotics, *n* (%)	57 (58.8)	23 (67.6)	0.600
Treatment with fluconazole, *n* (%)	51 (52.6)	10 (29.4)	0.019

*ICU* intensive care unit, *CVC* central venous catheter.

**Table 4:** Factors associated with 30-day mortality by multivariate analysis

**Variable**	**Odds ratio**	**95% Confidence interval**	***P*-value**
Complicated abdominal surgery	1.678	0.802–3.510	0.169
Presence of CVC	4.177	1.698–310.278	0.002
*C. tropicalis*	1.705	0.809–33.593	0.161
Treatment with fluconazole	0.523	0.247–31.110	0.091

*CVC* central venous catheter.

## Discussion

In recent years, some studies have reported that the incidence of candedimia is increasing in many hospitals around the world. Our data show that in our hospital the incidence of candidemia has increased steadily in the past three years in parallel with medical technological advances. The incidence is somewhat higher than that reported for centers in Denmark (0.41 case per 1000 admissions) [19], Israel (0.50 case per 1000 admissions) [20], China (0.53 cases per 1000 admissions) [21], and much lower than that reported in Brazil (1.87 cases per 1000 admissions) [22]. The differences in candidemia rates between countries may reflect differences in demographic characteristics, variations in health care practice, patterns using blood cultures and long duration of antibacterial usage as well as the resistance situation.

Neonates and infants have historically been populations with some of the highest rates of candidemia [23-29]. In our study, BSI by *candida* species occurred more frequently among males, mainly in those over 65 years old. The reasons for the shift in burden from neonates to adults are likely multifactorial; some contribution may be due to changes in the prevalence of risk factors in the adult population. Increases in common risk factors such as ICU admission [30,31], or numbers of patients receiving immunosuppressive therapies [32] may have resulted in increase in the overall pool of patients at high risk for candidemia. The difference in the distribution of cases between genders could be related to the predominant presence of male patients in units where the use of invasive devices, broad-spectrum antibiotics, extensive surgical procedures, or advanced life support is frequently used.

Over the past 20 years, a shift towards non-*albicans Candida* species has been reported previously from the USA, Europe and Australia, although the precise pattern of causative species varies across countries [5]. The findings from our surveillance are partially supportive of these reports. We observed a significant predominance of non-*albicans Candida* species (76.7%), with *C. tropicalis* being the most common isolate (28.6%), followed by *C. albicans* (23.3%), *C. parapsilosis* (19.5%) and *C. glabrata* (8.3%). Traditionally, *C. tropicalis* has been the second most common *Candida* species recovered from blood [33]. Similarly, as previously reported at a hospital in China, *C. albicans* (57.8%) continued to play a dominant role in candidemia, followed by *C. tropicalis* (12.8%) [34]. In our report, *C. tropicalis* surpassed the other *Candida* species to become the most common species, we recognize that this finding may simply be a result of the small sample size, and the further studies with larger sample size are needed to verify it. It is worth mentioning that eight *C. pelliculosa*, seven *C. famata* and two *C. haemulonii* isolates were recovered during the study period, suggesting the diversity of the etiologic agents. ARTEMIS DISK Global antifungal surveillance also found an increase of the involvement of less-common *Candida* species during the 10.5-year study period and showed that species identification is important to diagnosis and surveillance [35].

As already reported [36]. non-*albicans Candida* species were predominant in patients with haematological malignancies (83.3%), a finding in accordance with the global results of the ECMM survey [7]; moreover, *C. tropicalis* was frequently isolated in this group of patients, the prevalence of *C. tropicalis* among patients with haematological malignancies is consistent with previous reports [19].

The antifungal susceptibility patterns revealed that voriconazole has excellent in vitro activity overall against *Candida* species. Successful salvage therapy with voriconazole for the treatment of candidemia in patients intolerant or refractory to other antifungal agents has been reported [37]. Some studies showed that voriconazole may be a suitable agent for salvage therapy of invasive candidiasis, even in the setting of previous azole exposure and *C. krusei* infection [36,38]. Most publications on antifungal resistance over the past 10 years have been concerned with resistance to triazole antifungals, especially fluconazole, itraconazole, and ketoconazole. For ketoconazole, it showed the exsitence of a sensitivity of 38.1% of strains studied, lower than the other three azoles. Ketoconazole resistance was observed in 50% of *C. albicans* and *C. parapsilosis*, respectively. Itraconazole resistance was observed for 30.5% of all *Candida* species and was also highest among *C. glabrata* (90.9% resistance), this sensitivity decline was similar to that previously published in Iowa Organisms Study [39]. In recent years, resistance to azole antifungal agents among *Candida* spp. is still uncommon. In Iceland, 97.3% of the *Candida* spp. isolates tested were susceptible to fluconazole [13]. A similar data has been reported from North India [40]. In contrast to other reports, antifungal resistance was a notable finding in our study and was mainly restricted to fluconazole. Our proportion of fluconazole-resistant isolates (53.9%) was higher than the rates observed with spain (9.8%) [41]. We propose two potential reasons, first, the number of isolates in this study is still not high enough, therefore the rate of azole resistance is higher than clinical reports. Second, the increasing use of fluconazole as antifungal therapy leads to a reduction in susceptibility and the appearance of resistant strains. Some reportes have demonstrated that candidemia due to non-*albicans* species increased and that was apparently correlated with an increasing use of azoles for prophylaxis or empirical treatment [42], in our study, almost all patients received empirical therapy, 65(45.9%) patients were treated with fluconazole, followed by voriconazole (18.8%), and caspofungin (11.3%). For the latter reason, it would be convenient to carry out antifungal susceptibility studies in order to establish the in vitro activities of antifungal agents against local isolates and also to detect shifts toward resistance as early as possible. When analyzed by species, apart from the intrinsically fluconazole-resistant species (*C. krusei*), the highest rate of resistance to fluconazole was for *C. glabrata* and *C. tropicalis* (more than 50%), which was consistent with other studies in whom the greatest resistance to fluconazole also showed *C. glabrata* (36%) [41]. Furthermore, discrepancies in vitro susceptibility to FLC rate of different strains studied in this single-center study could be associated to differences in species distribution of isolates tested in this study.

In the present study we decided to analyze prognostic factors in patients with candidemia, we found that complicated abdominal surgery, presence of CVC, neutropenia, candidemia due to *C. tropicalis*, poor treatment with fluconazole were predictors of mortality in the univariate analysis. Of all the variables significantly associated with mortality in the univariate analysis, presence of CVC was the only predictive factor of mortality in the multivariate analysis. This is in agreement with the findings of a hospital population-based surveillance study [43]. In a study of pediatric candidemia, Candidemia-associated mortality has been found to be 31-72% [44]. Similarly, the crude 30-day mortality rate in our study was 26% for all patients with candidemia, which is in concordance with rates reported from Chinese tertiary-care centers, ranging from 26.4% to 33.3% [21,34]. A possible explanation for the relatively low mortality of nosocomial candidemia is the increase in empirical and/or pre-emptive use of fluconazole for presumed invasive candidiasis. Furthermore, although the hospital is a tertiary care hospital it rarely has patients with solid organ or bone marrow transplants. Thus, on average the patients in our hospital are less sick than in other hospitals. In our study, *C. tropicalis* was the species associated with the highest mortality rate (50%), besides being raised among the elderly, mortality is particularly high among patients with cancer (66.7% for haematological malignancies) as reported previously [33,45,46]. Certainly the severity of the underlying medical conditions has greatly influenced the crude mortality rate in these patient populations.

## Conclusions

This report shows that candidemia is a significant source of morbidity in Nanjing, with a substantial burden of disease, mortality, and likely high associated costs. We concede that there are several potential limitations to the present study. First, this is a single-center study. Our conclusions might be influenced by the local ecology, management practices, infections control policy, or our own susceptibility patterns. Second, our analysis was restricted to adult patients. Therefore, our conclusions cannot be extrapolated to pediatric populations. Finally, the results of the multivariate analyses might be influenced by the sample size and the number of variables included in the models. However, it would be of immense value to establish surveillance of candidemia to develop and evaluate prevention strategies and to monitor for changes in incidence and resistance.

## Competing interests

The authors declare that they have no competing interests.

## Authors’ contributions

FQL conceived, coordinated and designed the research; CFM performed the experiment, contributed to the acquisition, analysis and interpretation of data and drafted the manuscript. LNS, YAH, YW and MH participated in *Candida* spp. identification; QQK participated in sample collection and data acquisition. All the authors have read and approved the final manuscript.

## Pre-publication history

The pre-publication history for this paper can be accessed here:

http://www.biomedcentral.com/1471-2334/13/337/prepub
